# RhythmicDB: A Database of Predicted Multi-Frequency Rhythmic Transcripts

**DOI:** 10.3389/fgene.2022.882044

**Published:** 2022-06-14

**Authors:** Stefano Castellana, Tommaso Biagini, Francesco Petrizzelli, Andrea Cabibbo, Gianluigi Mazzoccoli, Tommaso Mazza

**Affiliations:** ^1^ Bioinformatics Unit, Fondazione IRCCS Casa Sollievo Della Sofferenza, San Giovanni Rotondo, Italy; ^2^ Department of Biology, Centro di Bioinformatica Molecolare, University of Rome “Tor Vergata,”, Rome, Italy; ^3^ Department of Medical Sciences, Division of Internal Medicine and Chronobiology Laboratory, Fondazione IRCCS Casa Sollievo Della Sofferenza, San Giovanni Rotondo, Italy

**Keywords:** periodicity, gene expression, circadian regulation, time-course data, clock genes

## Abstract

The physiology and behavior of living organisms are featured by time-related variations driven by molecular clockworks that arose during evolution stochastically and heterogeneously. Over the years, several high-throughput experiments were performed to evaluate time-dependent gene expression in different cell types across several species and experimental conditions. Here, these were retrieved, manually curated, and analyzed by two software packages, BioCycle and MetaCycle, to infer circadian or ultradian transcripts across different species. These transcripts were stored in RhythmicDB and made publically available.

## Introduction

The molecular clockwork of living organisms, common to diverse kingdoms of life, comprising Archaebacteria, Eubacteria, Protista, Fungi, Plantae, and Animalia, is a highly conserved endogenous biological timer that allows anticipation and adaptation to cyclic environmental transitions, provides competitive advantages for species survival and regulates cellular responses. The biological clock works through a complex interplay between genomic regulation, transcriptional activation-repression, and post-translational cellular processes (Hurley, Loros and Dunlap, 2016). Time-related variations of gene expression mainly show 24 h (circadian) rhythmicity, even if a few hundred genes are featured by the first (12 h) and second (8 h) harmonics of circadian rhythmicity, thereby showing correlation to light-to-dark/warm-to-cold transition phase and stress-response processes and metabolism, respectively (Lloyd and Murray, 2005; Hughes et al., 2009).

Over the past 3 decades, an ever-growing number of studies reporting results of high-throughput time-series experiments were published and accumulated many multi-omics datasets. Most data are accessible from the EBI-ArrayExpress (Parkinson et al., 2007) and NCBI-GEO (Barrett et al., 2013) databases. Their wide availability pushed the development of specialized computational resources to store time-series analysis of transcriptomes and associate biological functions (Supplementary Table S4). **CircaDB** (Pizarro et al., 2013) provides information on mouse and human time-series transcript profiles with 24 h periodicity. It is equipped with a simple query interface by which users can define filters, query search modality, and target tissues. Plots of time-course expression levels and statistical parameters for each queried gene are returned. Rhythms can be identified through three different methods, i.e., *JTK* (Hughes, Hogenesch and Kornacker, 2010), *Lomb Scargle* (Glynn, Chen and Mushegian, 2006), and *de Lichtenberg* (de Lichtenberg et al., 2005). CircaDB’s latest update notice falls back to 2014. Although raw data are downloadable, plots and processed results for about 3,000 cycling genes are not. **SCNseq** (Pembroke et al., 2015) is an R Shiny app that reports the circadian expression profiles of genes expressed in the mouse suprachiasmatic nucleus. For each gene, expression plots, i.e., read counts per timepoints, expression and oscillation measures, e.g., FPKM, Peak phase, and adjusted *p*-values are available. Analysis of oscillation was also performed for lincRNA molecules. The **DIURNAL** web resource (Mockler et al., 2007) is devoted to studying circadian gene expression in plant model organisms, offering a simple interface for querying several experimental conditions, loci, or probe accession numbers. Queries return graphical representations of time-series gene expression levels and the best-fitting harmonic model as calculated by the HAYSTACK method. Bulk download of *Arabidopsis thaliana, Oryza sativa, Glycine max, Populus trychocarpa, Brachypodium distachyon* normalized gene expression data, and probe-specific best models were also made publicly available. **CGDB** (Li et al., 2017) represents the most complete and updated tool for circadian genes, containing information on validated and predicted (by orthology) genes in hundreds of species. The tool can be queried using gene names, functions, or species names and retrieves curated data, such as oscillation period, phase, evidence level, tissue/cell context, experimental conditions, and associated publications. Initially, 27,964 genes of 25 distinct species, from *Drosophila melanogaster* to *Homo sapiens* to *Glycine max*, were reported to possess specific oscillatory expression patterns. Around 44,000 more genes for 148 other species were tagged as “putative circadian genes” by orthology. **CircadiOmics** (Patel et al., 2012) integrates different transcriptomic, proteomic, and metabolomic datasets to allow users to retrieve expression levels and dosage variation for oscillating metabolites, transcripts, and proteins. The whole dataset cannot be downloaded to date, and the query interface is not of immediate usage. Similarly, **CirGRDB** (Li et al., 2018) contains public transcriptomic and epigenomic datasets of human and mouse tissues. These were utterly re-analyzed, and rhythmicity was detected by the LSPR algorithm (Yang, Zhang and Su, 2011). LSPR was also used to predict oscillatory binding events and histone modifications among the different experimental conditions. Furthermore, rhythmicity was evaluated for non-coding RNA such as enhancer RNAs and RNA-edited molecules. Expression and regulatory data are also retrievable as plain text files.

**RhythmicDB** collects the results of a recent effort of homogeneous re-analysis of 87 time-course gene expression datasets derived from 48 publicly available experiments across 19 different species. Unlike other tools, oscillatory genes were identified using two distinct algorithms and were sought in carefully chosen datasets from which data of mutant, treated, or unhealthy strains were filtered out. In addition, algorithms were also used to detecting ultradian rhythmicity across the selected time-series data. Datasets and resulting predictions were made available freely from http://rhythmicdb.css-mendel.it.

## Methods

From October 2017 to December 2019, we queried the EBI ArrayExpress database with the terms: “circadian,” “time,” “clock,” “rhythm,” and “ultradian.” We then manually screened the matching results and selected only the datasets for which we could retrieve: preprocessed/normalized values of gene-wise expression time series; information about the sample origin and type (e.g., mutational state, treatments, health/disease state); at least four sampling points, i.e., expression time-series with maximum 6 h sampling interval and minimum 24 h duration. For each selected experiment, we i) downloaded the data files (“. *GSM files*”) corresponding to the expression values of control samples only for each time point, ii) merged them into expression matrices, and iii) made them available from the RhythmicDB website’s download section. GSM files associated with mutant/knock-out samples were discarded, while probes or sequence functional annotations were taken from the experiment-specific Array Design File. If an experiment consisted of multiple conditions (e.g., tissue, entrainment method, different RNA extraction protocol), distinct matrices were generated whenever different tissues, strains, or entrainment cycles were available for the same experiment (Supplementary Table S1). **MetaCycle** (Wu, et al., 2016) ver. 1.2 (MC) and **BioCycle** (Agostinelli, et al., 2016) ver. 0.9.3 (BC) were then run on these datasets, looking for ultradian (7–9 h and 10–14 h) and circadian (20–28 h) genes.

MetaCycle was installed in a R (R Core Team, 2021) 3.5 environment and run through the following command brueprint:

meta2d (infile, outdir = res, filestyle = “txt”, timepoints = points, minper = T1, maxper = T2, cycMethod = c (“LS,” “JTK”), outputFile = TRUE, adjustPhase = “predictedPer").

where “infile” is the input dataset; “timepoints” indicates the number of time points within the “infile”; “minper"/"maxper” constrain the minimum and maximum lengths for the oscillatory periods of interest; “cycMethod” indicates which MC algorithms should be used among “JTK” and “LS”; “adjustPhase” indicates that the inferred phase needs to be adjusted with the predicted period length. MC calculates a set of oscillatory properties for each probe, i.e., the period, adjusted phase, and amplitude. Estimates are provided with raw and adjusted *p*-values (Supplementary Table S1). We purposely did not consider the ARSER method integrated with MetaCycle since it cannot manage replicated time points or time-series taken at time intervals of different lengths.

BioCycle was run with the following command line:

Rscript ∼/BioCycle_0.9.3/BioCycle.R -i ∼/E-GEOD-XXXX_input.txt–o ∼/E-GEOD-XXXX/T7_T9/-s T1 -e T2

“E-GEOD-XXXX_input.txt” is an expression matrix file; “T1” and “T2” indicate the period interval for which the algorithm is trained. Significantly, we did not add any “GroundTruth” column in the input dataset because we had no prior knowledge about which probes were periodic or not. The tested oscillatory periods were: T1 = 7 AND T2 = 9 (7–9 h, ultradian); T1 = 10 AND T2 = 14 (10–14 h, ultradian); T1 = 20 AND T2 = 28 (20–28 h, circadian) (Supplementary Table S2).

A total of 522 independent runs were carried out (i.e., 87 experiments × two methods × 3 ″T1/T2” parameters). According to either of the tools, only the probes or sequenced genes/transcripts that oscillated significantly (i.e., *p*-value ≤ 0.05) were considered. They were annotated with information about the original experiment id, organism, period, and other sequence-related information. Details are given in Supplementary Tables S1, S2. In particular, the “Sequence.Info” field contains additional annotations, as provided by the Array Design Files, or genomic/transcriptomic annotations, if provided by the original datasets.

A mobile-friendly web interface was implemented in Vue. js ver. 2.6 for these data. It allows searching for oscillating transcripts through one or all of the 19 available species and eventually restricting the matching results to only those with a specified significance level or circadian or ultradian oscillation periods. This can be equally obtained for MC and BC. The output page recapitulates the organism, period range, and the transcript name of a searched molecule. Moreover, it displays the calculated p/q-values, the inferred oscillation period, lag, and amplitude values. RhythmicDB has a running instance of MongoDB ver. 4.4 behind it that currently contains and serves around two million records to the client through an Express. js ver. 4.17 middleware.

## Analysis

The analyses were conducted using MC and BC because of their computational efficiency, configuration and usage friendliness, comprehensibility of the output files, and resilience to missing values and low sampling rates. We processed a total of 2,073,343 probe sets across the 87 selected datasets. 173,505 (8.4%) and 401,886 (19.4%) showed significant oscillations according to the two software packages. While the usage of stringent statistical cutoffs would have certainly reduced the possibility of false-positive results, as observed in a previous benchmark analysis (Mei et al., 2020), we have set a relaxed *p*-value threshold to the canonical 0.05 value, intending to provide a maximal set of oscillating transcripts, eventually filterable in subsequent analysis stages. The web interface indeed allows further filtration by more stringent p/q-value thresholds. The outcome of the different significance thresholds for the two approaches is provided in Table 1.

**TABLE 1:** Number and relative proportion of oscillating transcripts, calculated for each method and tested periodicities.

Periodicities	MC	BC	Category
All	173,505	401,865	—
T7_T9	29,580 (17%)	71,543 (17.8%)	total
T10_T14	20,605 (12%)	107,197 (26.7%)	
T20_T28	123,320 (71%)	223,125 (55.5%)	
All	121,745	119,516	—
T7_T9	20,003 (16.4%)	17,832 (15.2%)	common
T10_T14	14,146 (11.6%)	14,088 (11.8%)	
T20_T28	87,596 (71%)	87,596 (73%)	
All	20,976	32,865	—
T7_T9	-	52 (0.2%)	stringent
T10_T14	165 (0.8%)	137 (0.4%)	
T20_T28	20,811 (99.2%)	32,676 (99.4%)	

Numbers are stratified per category (total: number of significant, i.e., *p*-value ≤0.05, circadian and ultradian transcripts over the total numbers; common: common transcripts between MC and BC; stringent: as ‘total’ but considering q-value ≤0.05).

While the proportion of transcripts oscillating with 7–9 h periodicity is similar (∼17%) between MC and BC, differences emerge for the other two tested periodicity values (Table 1, cf. category “total,” i.e., the proportions of circadian and ultradian transcripts over the total numbers, for each tool). Approximately 120,000 probe sets or transcripts were predicted to oscillate with the same periodicity, 87,596 of which significantly. Proportions differed slightly between MC and BC, i.e., 71 and 73%, respectively. The difference can be ascribed to the fact that certain transcripts were considered to oscillate with two period intervals (7–9h and 10–14 h) by one method and with a single period by the second one.

The application of two independent inference methods allowed us to achieve higher confidence in identifying oscillating transcripts. The list of known circadian rhythm-associated transcripts across different experiments is provided in Supplementary Table S3. Of note, 35 circadian transcripts were systematically found in 46 datasets for 11 different species **(** *A. thaliana, O. tauri, S. elongatus, A. aegypti, A. gambiae, D. melanogaster, D. rerio, H. sapiens, M. musculus, R. norvegicus, P. troglodytes*). To summarize, 213 known circadian genes were retrieved across different experiments. BC detected 105 transcripts, MC 21, and both 87 (40%). This highlights how inference methods impact the identification of circadian rhythmicity, an issue that has been intensely discussed (Hughes et al., 2017) and evaluated (Wu et al., 2014; Mei et al., 2020).

Focusing on ultradian oscillations (7–9 h), 67% of all significantly oscillating transcripts retrieved by MC (20,003 in 29,580) were also found by BC. Similar proportions resulted when studying 10–14 h period oscillating genes (14,146 out of 20,605). BC predicted many more oscillating transcripts; then, their intersection decreased to 13% for 10–14 h and 25% for 7–9 h periodicity). Increasing the statistical stringency (q-value ≤0.05), 99% of detected oscillating genes were circadian, and only ∼1% exhibited an ultradian period. Similar proportions apply to BC (details in Table 1).

We also reported the amount and relative proportions of 7–9 h, 10–14 h, and 20–28 h, significantly oscillating transcripts across all investigated datasets (Supplementary Table S2). The high variability of these results is partly explained by the fact that rhythms are strictly species- and tissue-specific (Mure et al., 2018) and partly by the different experimental protocols, entrainment strategies, sampling intervals, experiment duration, and quantification/normalization methods used to generate the data that we collected in RhythmicDB. As expected, 20–28 h circadian periodicity was predominantly frequent among all species, although their proportions varied largely across different experiments and tissues. Conversely, the proportion of ultradian transcripts was generally negligible across all datasets. Although the detection of ultradian transcripts is, in fact, biologically meaningful (Westermark and Herzel, 2013; Zhu, Dacso and O'Malley, 2018), high-frequency assessment, i.e., at least 2 h time intervals, is technically challenging. It requires denser sampling intervals than the prevalent setups, appropriate experimental designs, and ad-hoc computational techniques. While the bioinformatics community has made great efforts in this direction (Zhu et al., 2017; Veen and Gerkema, 2017), further research is still needed.

Until now, several bioinformatics tools have been conceived for the detection of biological rhythms and storage of their results (de Lichtenberg et al., 2005; Glynn, Chen and Mushegian, 2006; Mockler et al., 2007; Hughes, Hogenesch and Kornacker, 2010; Yang and Su, 2010; Yang, Zhang and Su, 2011; Straume, 2004; Thaben and Westermark, 2014; Agostinelli, Ceglia, Shahbaba, Sassone-Corsi, et al., 2016; Wu et al., 2016; Abhilash and Sheeba, 2019; Singer and Hughey, 2019; Carlucci et al., 2020; De Los Santos et al., 2020; Montroya et al., 2020; Ness-Cohn et al., 2020). Nevertheless, we have implemented and released RhythmicDB to include the datasets elaborated in this study because a bioinformatic tool permitting data integration of circadian with ultradian oscillating transcripts across multiple species was lacking. Therefore, RhythmicDB should not be considered an alternative to the above databases but a complementary tool to inquire for ultradian rhythmic transcripts as well. These transcripts were not collected from the literature or public resources (e.g., Uniprot or Pfam), as, e.g., CGDB does, but were obtained at the end of an essential preprocessing step, which included downloading raw gene expression data from EBI ArrayExpress and NCBI GEO, dropping mutant or unhealthy samples and pruning datasets with biased sampling times or duration. At the end of this process, transcripts were called using two renowned packages for rhythmicity detection. Hence, several databases above, with CGDB in the lead, are of mention as the first and primary references to explore circadian transcripts, as they come from literature. On the contrary, RhythmicDB can be helpful to explore the natural variability of molecular oscillations across species/tissues; corroborate or not the circadian rhythmicity of transcripts by comparing the MetaCycle/BioCycle results; suggest new possible, i.e., unreported by the literature, circadian and ultradian genes.

In perspective, we plan to integrate new annotation sources, e.g., updated gene symbols, reference transcript accession numbers, and Gene Ontology terms, whenever possible. In fact, the species included in RhythmicDB are not equally well-annotated, with some being subjected to frequent data modification or replacement and suffering from data inconsistency. Moreover, we will periodically update RhythmicDB so as to include new gene expression datasets that MC and BC can process.

## Data Availability

The original contributions presented in the study are included in the article/Supplementary materials, further inquiries can be directed to the corresponding authors.
