# Supplementary material for: RhythmicDB: A Database of Predicted Multi-Frequency Rhythmic Transcripts
Source: Front Genet. 2022 Jun 14;13:882044. doi: 10.3389/fgene.2022.882044 (PMC9237250; doi:10.3389/fgene.2022.882044)
Supplement: Supplementary file 1 [file Table2.DOCX]

Supplementary Material

# Supplementary Data

# All raw data files for the selected experiments (listed in Supplementary Material 2, Table S1) are available at: <http://rhythmicdb.css-mendel.it/rhythmicdb_raw_experimental_datasets.rar>. Each folder is named with the corresponding EBI ArrayExpress Identifier and contains GSMNNNN *.txt files with expression values for each single time point; Array Design File (*.adf.txt) containing probe annotations for array experiments; sample table (*.sdrf.txt), a tabular description of sample features. Expression input matrices for MetaCycle and BioCycle are available at: <http://rhythmicdb.css-mendel.it/rhythmicdb_raw_input_datasets.rar>. Files are named according to their corresponding EBI ArrayExpress identifier: the first column reports sequence name or accession or probe id; flanking columns show expression values per collected time point. These are ordered from 0 to the last point (total duration of the experiment); for experiments with point replicates, replicate time points are clustered together.

Tools were downloaded from:

**MetaCycle**: <https://cran.r-project.org/web/packages/MetaCycle/index.html>

**BioCycle**: <http://circadiomics.igb.uci.edu/static/downloadables/BioCycle.tar.gz>

# Supplementary Figures and Tables

## Supplementary Table 1

| **#** | **Field** | **Description** | **Example** |
| --- | --- | --- | --- |
| 1 | Dataset | - ArrayExpress or GEO accession id; an underscore evidences possible subsets | - “E-GEOD-17627_IL” |
| 2 | Species | - Symbol for the species name | - “Mmu” |
| 3 | Period | - Tested oscillatory period, indicated as a time interval. | - “T7_T9” |
| 4 | Sequence.info | - Any gene, protein ID, functional annotation or description associated to a probe. | - “RND1;NM_014470” |
| 5 | Element | - Probe number or accession as retrieved by each single primary dataset. | - “13496995” |
| 6 | meta2d_pvalue | See PMID:15075390 and  https://cran.r-project.org/web/packages/   - MetaCycle/vignettes/implementation.html   for output description. | - Floating or integer number |
| 7 | meta2d_BH.Q |  | Floating or integer number |
| 8 | meta2d_period |  | Floating or integer number |
| 9 | meta2d_phase |  | Floating or integer number |
| 10 | meta2d_Base |  | Floating or integer number |
| 11 | meta2d_AMP |  | Floating or integer number |
| 12 | meta2d_rAMP |  | Floating or integer number |

**Supplementary Table 1.** Description and examples for the global output table for MetaCycle significantly (p-value ≤ 0.05) oscillating elements. Fields n. 1, 2, 5 – 12 are presented within the RhythmicDB output page; field 4 has been split into two sub-fields, “Gene info” and “Transcript info.”

## Supplementary Table 2

| # | **Field name** | **Description** | **Example** |
| --- | --- | --- | --- |
| 1 | Dataset | - ArrayExpress or GEO accession id; an underscore evidences possible subsets | - “E-GEOD-17627_IL” |
| 2 | Species | - Symbol for the species name | - “Mmu” |
| 3 | Period | - Tested oscillatory period, indicated as a time interval. | - “T7_T9” |
| 4 | Sequence.info | - Any gene, protein ID, functional annotation or description associated to a probe. | - “CASP3;NM_032991” |
| 5 | Element | - Probe number or accession as retrieved by each single primary dataset. | - “13496995” |
| 6 | P_VALUE | See PMID:29912458 and http://circadiomics.igb.uci.edu/help for output description. | Floating or integer number |
| 7 | Q_VALUE |  | Floating or integer number |
| 8 | PERIOD |  | Floating or integer number |
| 9 | LAG |  | Floating or integer number |
| 10 | AMPLITUDE |  | Floating or integer number |
| 11 | AVG_REPS_SCATTER |  | Floating or integer number |
| 12 | REPS_SCATTER |  | Floating or integer number |

**Supplementary Table 2.** Description and examples for the global output table for BioCycle significantly (p-value ≤ 0.05) oscillating elements. Fields n. 1, 2, 5 – 12 are presented within the RhythmicDB output page; field 4 has been split into two sub-fields, “Gene info” and “Transcript info.”
